# A new species of *Claviramus* (Annelida, Sabellida, Sabellidae) from the Ariake Inland Sea, Kyushu, Japan

**DOI:** 10.3897/zookeys.880.36281

**Published:** 2019-10-14

**Authors:** Eijiroh Nishi, Katsuhiko Tanaka, María Ana Tovar-Hernández

**Affiliations:** 1 College of Education, Yokohama National University, Hodogaya, Yokohama 240-8501, Japan Yokohama National University Hodogaya Japan; 2 Department of Marine Biology, School of Marine Science and Technology, Tokai University, 3-20-1, Orido, Shimizu-ku, Shizuoka-shi, Shizuoka 424-8610, Japan Tokai University Orido Japan; 3 Universidad Autónoma de Nuevo León, Facultad de Ciencias Biológicas, Laboratorio de Biosistemática, San Nicolás de los Garza, Nuevo León, México Universidad Autónoma de Nuevo León San Nicolás de los Garza Mexico

**Keywords:** fan worms, *
Jasmineira
*, Polychaeta, soft bottoms, taxonomy

## Abstract

A new species of the sabellid polychaete genus *Claviramus* Fitzhugh, 2002, is described from Ariake Inland Sea, Kyushu, Japan. *Claviramus* is a small genus, composed of three species worldwide. Its distinctive feature is the presence of foliaceous flanges at the distal ends of the radioles. *Claviramus kyushuensis***sp. nov.** here described is characterized by the presence of a glandular ridge on chaetiger 2, glandular shields on the abdomen, thoracic uncini bidentate, and the presence of a short, distal filament in some radioles. A key and a comparative table of diagnostic characters for species of *Claviramus* are provided.

## Introduction

Japanese waters are represented by approximately 40 species of sabellid polychaetes (Nishi et al. 2017). Among them, eight species from soft bottoms belonging to plesiomorphic genera *Chone* Krøyer, 1856, *Dialychone* Claparède, 1870, *Jasmineira* Langerhans, 1880 and *Paradialychone* Tovar-Hernández, 2008, have been reported (Nishi et al. 2009). In this study, a new species of *Claviramus* Fitzhugh, 2002 is described from Ariake Inland Sea, Kyushu, Japan. It was found co-occurring with *Jasmineira kikuchii* Nishi, Tanaka, Tovar-Hernández & Giangrande, 2009.

The sabellid genus *Claviramus* is currently composed of three species worldwide. *Claviramus candelus* (Grube, 1863), the type species of the genus, was originally described as *Sabella candela* Grube, 1863, from the northern Adriatic Sea, but Langerhans (1884) transferred it to the genus *Jasmineira*. *Claviramus oculatus* (Langerhans, 1884) was described as *Jasmineira oculata* Langerhans, 1884, from Madeira. Cochrane (2000) redescribed both species within *Jasmineira* in detail based on type and additional specimens. Fitzhugh (2002) established the genus *Claviramus* based on the presence of prominent foliaceous flanges at the distal ends of the radioles, and transferred *J. candelus* and *J. oculatus* to *Claviramus*. The third known species, *Claviramus grubei* Fitzhugh, 2002, was described from Thailand, Andaman Sea. A thorough revision and synthesis of these three species was provided by Cochrane (2000) and Fitzhugh (2002).

## Materials and methods

Specimens were measured to record width of the middle of the thorax, trunk length (chaetiger 1 or collar to pygidium), radiolar crown length, number of radiolar pairs, number of thoracic and abdominal segments, and presence of gametes. The diagnosis and a full description of the new species were based on the holotype, with variation in the paratypes indicated in parentheses. The thoracic and abdominal glandular pattern was revealed by staining the worms with methyl green. Parts of thorax and abdomen of one paratype CBM-ZW 1124 were observed on the scanning electron microscope JSM-6500 at the Yokohama National University. Digital photographs were taken with an attached Canon EOS Rebel T7i digital camera. Type materials were deposited at the Natural History Museum and Institute, Chiba, Japan (catalogue code CBM-ZW) and at the Colección Poliquetológica, Universidad Autónoma de Nuevo León (catalogue code UANL). A key and a comparative table of diagnostic characters for species of *Claviramus* are also included; the information is as complete as available based on original descriptions and redescriptions provided by Cochrane (2000) and Fitzhugh (2002).

## Taxonomic account

### Order Sabellida Latreille, 1825

#### Family Sabellidae Latreille, 1825

##### Genus Claviramus

Taxon classificationAnimaliaSabellidaSabellidae

Fitzhugh, 2002

A4E1559C-876F-59E6-9E47-4FC784EF4411

Claviramus Fitzhugh, 2002: 412, 414–415.

###### Type species.

*Sabella candela* Grube, 1863.

##### Claviramus kyushuensis sp. nov.

Taxon classificationAnimaliaSabellidaSabellidae

2F355BA9-8140-58FA-92D4-5FEA0867A589

http://zoobank.org/AF7C503A-E9B9-4424-840B-191F1718015A

Figs 1 , 2 , 3

###### Material examined.

Ariake Sound, Kyushu, Japan, Stn 20D, 32°31.070'N, 130°14.037'E, 20 m depth, sandy mud bottoms, collected by dredge by K. Mori, 17 September 2005. **Holotype** CBM-ZW 1123, **Paratypes** CBM-ZW 1124-1126 (three paratypes: two complete, one lacking crown), UANL 8130 (three paratypes: two complete, one lacking crown).

###### Diagnosis.

**Figure 1.**
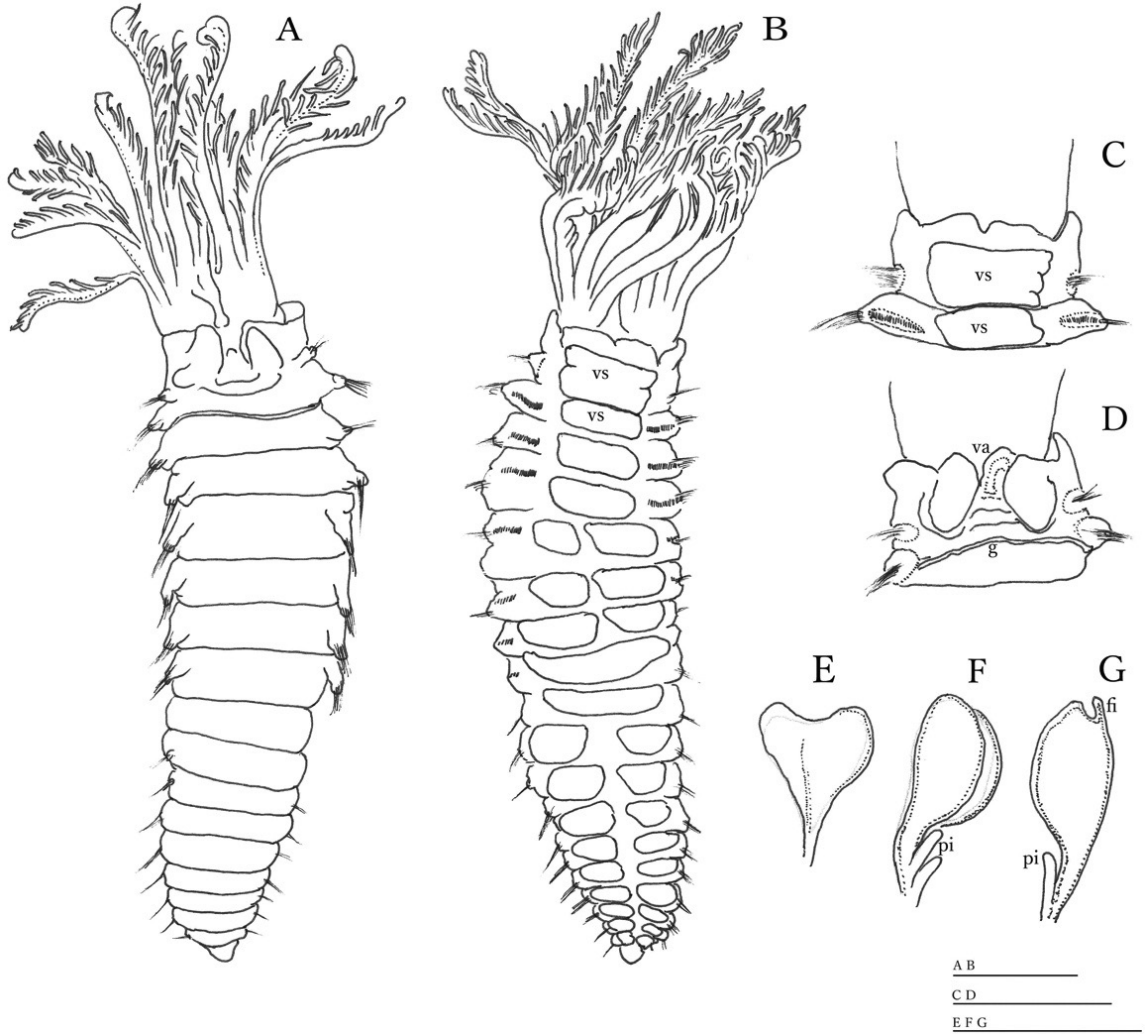
*Claviramus kyushuensis* sp. nov., holotype. **A** Body, dorsal view **B** same, ventral view **C, D** collar and second chaetiger, ventral and dorsal views, respectively **E–G** distal foleaceous flanges. Abbreviations: fi: filament, g: glandular ridge, pi: pinnule, va: vascular loop, vs: ventral shield. Scale bars: 0.5 mm (**A–D**), 0.2 mm (**E–G**).

**Figure 2.**
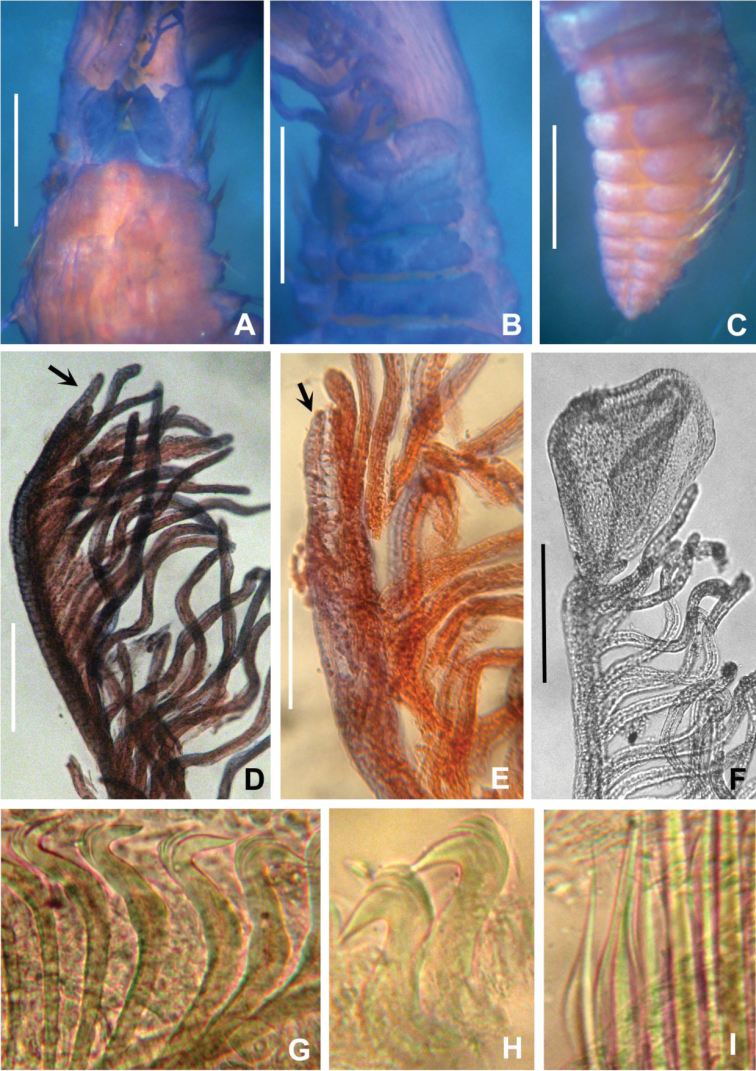
Paratype of *Claviramus kyushuensis* sp. nov. **A** Anterior thoracic segments, dorsal view **B** same, ventral view **C** posterior abdomen **D–F** radiolar tips **G** thoracic uncini **H** abdominal uncini **I** thoracic chaetae. Arrows in **D** and **E** point to radiolar tips, entire in **D**, broken in **E**. Scale bars: 0.5 mm (**A–C**), 150 um (**D–F**).

**Figure 3.**
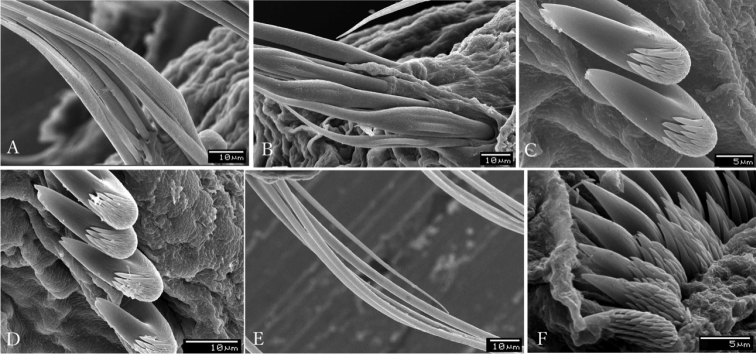
*Claviramus kyushuensis* sp. nov., scanning electron microscope images of chaetae and uncini. **A** Collar chaetiger with narrowly hooded chaetae **B** second thoracic chaetiger with superior group of narrowly hooded chaetae and inferior broadly hooded chaetae **C** uncini from seventh thoracic torus **D** uncini from fourth thoracic torus **E** elongate, narrowly hooded chaetae from eighth abdominal chaetiger **F** uncini from the seventh abdominal torus.

Subdistal ends of some radioles with lateral margins extended, thin, as foliaceous flanges (Figs 1E–G, 2F), some with a short, distal filament or cirrus (Fig. 1G). Glandular ridge on chaetiger 2 present. Abdominal shields well developed (Figs 1B, 2C). Dorsal pockets of collar present exposing large vascular loops (Fig. 1D). Anterior peristomial ring not extending beyond ventral collar margins. Ventral margin of collar with a shallow mid-ventral incision forming two discrete rounded lappets (Figs 1B, C). Thoracic tori not contacting shields (Fig. 1B). Thoracic uncini with tips of main fangs bifid (Fig. 3C–D).

###### Description.

Sabellid worm with eight thoracic (eight in all types) and ten abdominal chaetigers (9–16 in paratypes CBM ZW 1124-1126, UANL 8130). Trunk length 2.5 mm (1.6 mm in paratype CBM-ZW 1125, 3.2–4.7 mm in paratypes UANL 8130), body width 0.7 mm (0.3 mm in paratype CBM-ZW 1126, 0.5–1.3 mm in paratypes UANL 8130). Radiolar crown 1.1 mm length (1.3–2.1 mm in paratypes UANL 8130), with seven radioles in each branchial lobe (7–9 in paratypes UANL 8130).

Palmate membrane absent. Subdistal ends of some radioles with lateral margins extended, thin, as foliaceous flanges; overall shape oblong (Figs 1E–G, 2F) with a mid-ventral incision occupying a quarter of flange length; some tips with a short, distal filament (Fig. 1G). Other radioles with unflanged tips, filiform (Fig. 2D) or with broken tips (Fig. 2E). Largest pinnules located at 3/4 of radiole length (Fig. 2D). Radiolar eyes absent. Two pairs of ventral radiolar appendages, as long as half of radiolar crown length. Dorsal lips narrow, triangular, longer than wide. Ventral lips rounded, low. Dorso-lateral margins of collar fused to faecal groove; dorsal pockets present (Figs 1A, D, 2A); large vascular loops visible on dorsal pockets of collar (Fig. 1D); ventral sacs absent. Ventral margin of anterior peristomial ring as broadly triangular lobe, not extending beyond collar margins. Ventral collar margin with a shallow mid-ventral incision forming two discrete rounded lappets (Figs 1B, C, 2B). Lateral collar margins slightly oblique, with ventral margin slightly higher than dorsal. Thoracic and abdominal shields well developed (Figs 1B, C, 2C). Collar shield divided transversally into three nearly rectangular sections with lateral margins indented (Fig. 2B). A pair of white triangular glandular pads in the ventral side of collar, as lung-shaped. Shields from chaetigers 2 to 8 rectangular, broad, entire (Figs 1B, 2B). Abdominal shields forming two squares divided by faecal groove (Figs 1B, 2C). Narrow glandular ridge on chaetiger 2 present, most notorious laterally (Fig. 1A, D). Thoracic tori not contacting shields (Fig. 1B, C). Thoracic notopodial fascicles in chaetiger 1 as short as rows of narrowly hooded chaetae (collar chaetae) (Fig. 3A). Notopodial fascicles in chaetigers 2–8 with superior group of narrowly hooded chaetae and two inferior rows of broadly hooded chaetae (Figs 2I, 3B). Thoracic neuropodial uncini acicular (Fig. 2G); main fang bifid, surmounted by 5–6 rows of small equal-sized teeth (Fig. 3C, D), breast as a narrow swelling; handles very elongate (Fig. 2G). Abdominal neuropodial fascicles with one or two transverse rows of narrowly hooded chaetae (Fig. 3E). Abdominal notopodia with avicular uncini (Figs 2H, 3F); main fang surmounted by 7–9 rows of small teeth equal in size, occupying a half of the main fang length (Fig. 3F); breast well developed; handles short (Fig. 2H). Pygidium triangular without eyes neither cirrus (Figs 1B, 2C). Anus ventral. Tubes not preserved. Paratypes mature hermaphrodites with full-developed oocytes and sperm in thoracic and abdominal segments.

###### Etymology.

The specific epithet is named after type locality, Kyushu, Japan.

###### Remarks.

Among the species currently recognized in *Claviramus*, *C. kyushuensis* sp. nov., is unique by having a collar shield rectangular, divided transversally into three nearly equal-sized sections; a glandular ridge on chaetiger 2; abdominal shields well developed; main fang of thoracic uncini with bifid tips and the presence of a short, distal filament in some radioles.

*Claviramus grubei* has also a glandular ridge on chaetiger 2, a short mid-ventral incision of distal radiolar flanges and radiolar tip filaments, but it differs of *C. kyushuensis* sp. nov., by lacking abdominal shields (present in *C. kyushuensis* sp. nov.) (Table 1).

**Table 1.** Species of *Claviramus* from the world after Cochrane (2000) and Fitzhugh (2002).

Species name	Glandular ridge on chaetiger 2	Abdominal glandular shields	Mid-ventral incision of distal radiolar flanges	Ventral margin of collar	Ventral shield of collar	Main fang of thoracic uncini	Pygidial eyes	Type locality
*Claviramus candelus* (Grube, 1863)	Absent	Present	? (Short, less than 1/4 of the flange length, fide figure of Langerhans)	Even in height	Rectangular, entire	?	Present	Adriatic Sea
*Claviramus grubei* Fitzhugh, 2002	Present	Absent	Short, less than 1/4 of the flange length	With shallow mid-ventral incision	?	?	Absent	Thailand, Phuket Island
*Claviramus oculatus* (Langerhans, 1884)	Absent	Absent	Short, less than 1/4 of the flange length	With shallow mid-ventral incision	Rectangular, divided transversally into 2 areas (superior wider than inferior one)	?	Present	Madeira
*Claviramus kyushuensis* sp. nov.	Present	Present	Medium, 1/2 of the flange length	With shallow mid-ventral incision	Rectangular, divided transversally into 3 nearly equal sized sections with lateral margins indented	Bifid in frontal view	Absent	Ariake sound, Kyushu, Japan

*Claviramus kyushuensis* sp. nov., differs from *C. oculatus* and *C. candelus* mainly by lacking pygidial eyes (present in *C. oculatus* and *C. candelus*) and having a collar shield rectangular, divided transversally into three nearly equal-sized sections (entire in *C. candelus*, divided into two areas in *C. oculatus*) (Table 1).

In addition, SEM images used in this study reveals that tips of main fangs of thoracic uncini are bifid (Fig. 3C, D). This peculiarity has been only reported in *Amphicorina triangulata* López & Tena, 1999 by Cepeda and Lattig (2017). However, in *A. triangulata*, the presence of a large tooth above the main fang in the midline, followed by a third tooth offset from midline, and then followed by series of smaller teeth, is remarkable. In *Claviramus kyushuensis* sp. nov., all rows of teeth above the main fang are nearly equal-sized (Fig. 3C, D).

#### Key to species of *Claviramus*

**Table:** 

1	With ventral shields on abdominal segments	**2**
–	Without ventral shields on abdominal segments	**3**
2	Ventral margin of collar entire; glandular ridge on chaetiger 2 absent; with pygidial eyes	***C. candelus***
–	Ventral margin of collar incised; glandular ridge on chaetiger 2 present; without pygidial eyes	***C. kyushuensis* sp. nov.**
3	With pygidial eyes; glandular ridge on chaetiger 2 absent	***C. oculatus***
–	Without pygidial eyes; glandular ridge on chaetiger 2 present	***C. grubei***

## Discussion

*Claviramus* was erected based on the presence of prominent foliaceous flanges, at the distal ends of radioles (Fitzhugh 2002). However, in specimens reviewed here, these leaf-like structures are easily broken off and are present only in some radioles (other radioles have entire filiform tips revealed by presence of skeleton cells). Cochrane (2000) also showed broken radioles in some specimens belonging to *C. candelus*. Under this scenario, it is evident that many specimens were wrongly identified under *Jasmineira*.

However, *Jasmineira* and *Claviramus* may also distinguishable based on the presence of inferior thoracic bayonet notochaetae (absent in *Claviramus*), uncinial morphology (Fitzhugh 1989; Cochrane 2000) and presence of a breaking plane sensu Cochrane (2003) or abscission zone sensu Tovar-Hernández (2008). The abscission zone refers to crowns where there is a distinct point immediately above the radiolar bases, where the radioles become detached from the branchial basis.

## Supplementary Material

XML Treatment for Claviramus

XML Treatment for Claviramus kyushuensis
